# A meta-analysis of the relationship between circulating microRNA-155 and coronary artery disease

**DOI:** 10.1371/journal.pone.0274277

**Published:** 2023-04-13

**Authors:** Tao Ran, Jinyao Chen, Yu Cheng, Min Zhang, Min Mao, Rui Xiang, Zhong Zuo, Jing Chang, Baoru Han, Kanghua Ma

**Affiliations:** 1 The First Clinical College, Chongqing Medical University, Chongqing, China; 2 Department of Nursing, University Town Hospital Affiliated to Chongqing Medical University, Chongqing, China; 3 School of Public Health and Management, Chongqing Medical University, Chongqing, China; 4 Department of Cardiology, The First Affiliated Hospital of Chongqing Medical University, Chongqing, China; Baylor Scott and White, Texas A&M College of Medicine, UNITED STATES

## Abstract

**Objective:**

Coronary artery disease (CAD) is a leading cause of death worldwide. Many studies in China and abroad have reported an association between the expression level of *microRNA-155* and CAD; however, the results remain controversial. We aimed to comprehensively investigate this association based on a meta-analysis.

**Methods:**

We first systematically searched eight Chinese and English databases, including China National Knowledge Infrastructure, Wanfang, China Science and Technology Journal Database, PubMed, Web of Science, Embase, Google Scholar, and Cochrane Library, to identify studies concerning the relationship between *microRNA-155* levels and CAD published before February 7, 2021. The quality of the literature was assessed by the Newcastle–Ottawa Scale (NOS). Meta-analysis was performed using a random-effects model to calculate the standard mean difference with a 95% confidence interval (CI).

**Results:**

Sixteen articles with a total of 2069 patients with CAD and 1338 controls were included. All the articles were of high quality according to the NOS. The meta-analysis showed that the mean level of *microRNA-155* was significantly lower in patients with CAD than in controls. Based on subgroup analyses, the level of *microRNA-155* in the plasma of CAD patients and in acute myocardial infarction (AMI) patients was significantly lower than that in controls, whereas this level in CAD patients with mild stenosis was significantly higher than that in controls.

**Conclusion:**

Our study indicates that the expression level of circulating *microRNA-155* in patients with CAD is lower than that in a non-CAD group, suggesting a new possible reference index for the diagnosis and monitoring of patients with CAD.

## 1 Introduction

Coronary artery disease (CAD) is a heart disease caused by narrowing or blockage of the vascular lumen due to atherosclerosis of the coronary arteries, resulting in ischemia, hypoxia, and even necrosis of the myocardium. In recent years, CAD has been classified into chronic coronary artery disease and acute coronary syndrome according to clinical urgency and the importance of timely treatment. CAD is the most common chronic disease and is a primary cause of death, especially in Western countries. In recent decades, the incidence and mortality of CAD in Europe and the United States have shown a downward trend, though there has been an upward trend in China. Although the exact etiology and pathogenesis of CAD are still unclear, it is considered to be a polygenic and multifactorial disease influenced by environmental and genetic factors.

MicroRNAs are evolutionarily conserved endogenous noncoding single-stranded RNA small molecules composed of 20–23 nucleotides that are involved in regulating relevant gene expression. It has been shown that microRNAs play an important role in the pathological process of many diseases, including tumors and CAD [1]. MicroRNA-155 (also known as miR-155) is a subfamily of microRNAs located within the third exon of the noncoding transcript of human chromosome 21 and is a typical multifunctional miRNA [2]. In 2004, *microRNA-155* was first discovered to be related to the progression of lymphoma. Subsequently, meta-analyses have indicated that *microRNA-155* is also associated with other tumors, such as glioma and breast cancer. In addition to tumors, many observational studies in the past ten years have linked the association between the expression level of *microRNA-155* and CAD. For example, Faccini et al. found that expression of *microRNA-155* in patients with CAD was significantly lower than that in controls [3]. In contrast, Qiang Su et al. reported that *microRNA-155* expression in patients with CAD was significantly higher than that in controls [4]. These results show that the relationship between *microRNA-155* levels and CAD is controversial.

In the present study, we used meta-analysis to evaluate the association between the level of *microRNA-155* and CAD by quantitative and comprehensive statistics. To the best of our knowledge, this study is the first meta-analysis investigating this relationship.

## 2 Methods

We conducted the meta-analysis according to Meta-Analysis of Observational Studies in Epidemiology (Moose) guidelines.

### 2.1. Literature search strategy

We searched three Chinese and five English-language databases from inception to February 7, 2021, to identify studies on the relationship between CAD and expression of *microRNA-155*. The three Chinese databases are the Chinese National Knowledge Infrastructure (CNKI), China Science and Technology Journal Database (VIP) and Wanfang Database. The five English databases are PubMed, Web of Science, Embase, Google Scholar, and Cochrane Library. The keywords “coronary heart disease”, “coronary artery disease”, “coronary disease”,”coronary atherosclerosis”,”angina”, “myocardial infarction”, “ischemic heart disease”, “acute coronary syndrome”, “ischemic heart failure”, “ischemic cardiomyopathy” and “microRNA-155”, “miR-155”, “miRNA-155” were used in combination for full-text retrieval.

### 2.2. Study selection

Publications were selected if they met the following criteria: (1) observational study (including cross-sectional study, case–control study, cohort study) exploring the correlation between CAD and expression of *microRNA-155*; (2) the patients included were diagnosed with ≥ 50% stenosis in at least one major epicardial vessel evaluated by coronary arteriography (CAG) or computed tomography angiography (CTA); (3) for case–control studies, the control group was non-CAD patients or healthy people without a CAD development trend, and for cohort studies, the nonexposed group was non-CAD patients or healthy people without a CAD development trend; and (4) the included studies directly provided the mean level of *microRNA-155* and standard deviation (SD) in patients and controls or provided sufficient data to calculate these two measures. When multiple studies used the same population, we included the most recent article or that with the largest sample size.

Studies were excluded if (1) they were lectures, reviews, animal experiments, case reports or reviews; (2) the full text was unavailable; or (3) documentation of comorbidities, such as heart failure and myocarditis, was available.

### 2.3. Data extraction

Literature data extraction was performed independently by two researchers, and experienced researchers were consulted in cases of inconsistent results. Data were extracted using a predesigned form, which included (1) the basic characteristics of the literature, including first author, publishing year, country, confounding factors, and sample source, and (2) mean level of *microRNA-155* and SD in patients and controls. Different groups of the same study were considered as an independent study.

### 2.4. Quality evaluation

The Newcastle–Ottawa Scale (NOS) was used to evaluate the quality of each cohort study and case–control study, which included (1) the selection of case and control groups (appropriateness of case definition and diagnosis, continuity and representativeness of cases, selection of controls, definition of controls); (2) comparability; and (3) evaluation of exposure factors (methods of investigation and assessment of exposure, whether the survey method was the same for cases and controls, and the nonresponse rate). There are eight entries, and each entry has one point (two points for comparability): one point if it is met, and zero points if it is not, for nine points out of five, with five or more being high-quality articles.

The quality of each cross-sectional study was evaluated using the AHRQ scale. The scale consists of 11 items scored "yes", "no" or "unclear". The scoring method is 1 point for "yes", and 0 points for "no" or "unclear", and the total score for each item is summed to 0~11 points. The scores are 0~3 for low quality, 4~7 for medium quality and 8~11 for high quality. Medium- and high-quality literature was included.

### 2.5. Statistical methods

Statistical analysis was performed using Stata 16 software. We used Cochran’s Q test and the *I*^2^ statistic to assess heterogeneity between the studies. As there was great heterogeneity among the original studies included in this meta-analysis, we adopted the SMD method. Meta-analysis was performed using a random effects model when *I*^2^ ≥ 50% or a fixed-effects model when *I*^2^ < 50%. We conducted a meta-analysis for continuous variables and obtained the standard mean difference (SMD) with a 95% confidence interval (CI). We also conducted subgroup analyses by subtype of CAD, severity of CAD (stability of plaque, degree of stenosis, and number of diseased vessels), sample source (plasma or PBMCs), sample size (N > 100 or N < 100), and ethnicity (Asian or non-Asian). Publication bias was investigated by visual inspection of asymmetry in funnel plots. Begg’s test and Egger’s test were also performed to assess potential publication bias and small-study bias, respectively. We further conducted meta-regression to explore sources of heterogeneity. Sensitivity analysis was then performed to test the stability of the meta-analysis results. Tests of heterogeneity and bias were one-tailed, and a *p* value less than 0.10 was considered significant. A two-sided *p* value of 0.05 was considered statistically significant.

## 3 Results

### 3.1. Literature search results

**Fig 1:**
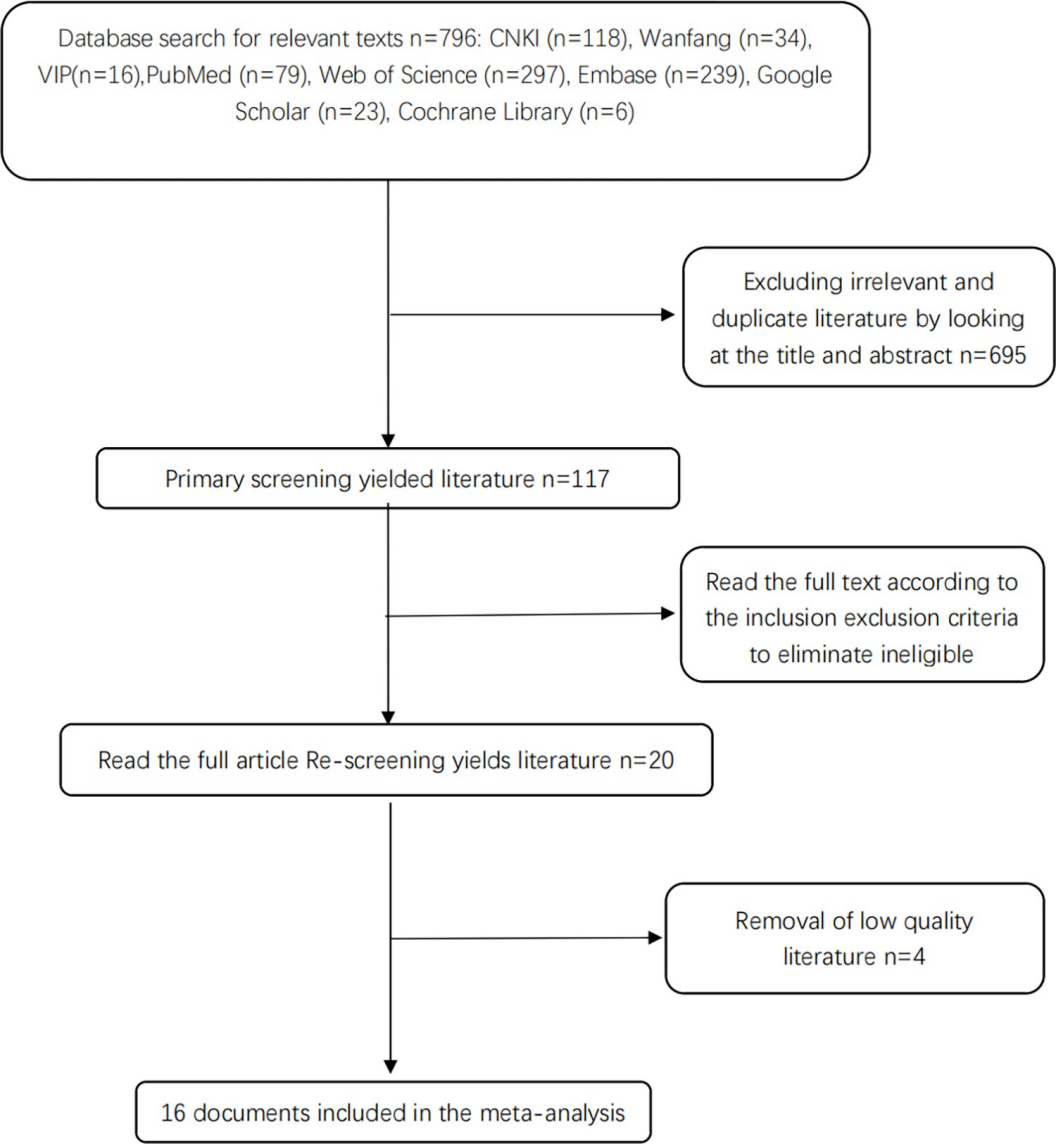
Literature search process and results.

The flow chart of the study selection is shown in **Fig 1**. A total of 168 articles in Chinese and 644 articles in English were retrieved. In total, 117 articles remained after the primary screening, and 16 were finally included in the meta-analysis after manual screening according to the inclusion and exclusion criteria.

### 3.2. Basic characteristics and quality of the literature

The basic characteristics of the 16 [1, 2, 4–17] included articles are shown in **Table 1**. All of them were case–control studies. The total number of included samples was 3407. Of these articles, ten are in Chinese and six in English. Fifteen articles were carried out in Asian countries and one in European countries, and all the articles were published after 2013. The quality scores of the included studies were all above 5.

**Table 1:** Basic characteristics and quality of the literature retrieved.

Study	Year	Ethnicity	Sample source	Subgroup of CAD	Control group		Case group		NOS
					Sample size	Result	Sample size	Result	
Jia Wu	2013	Asian	plasma	SAP	40	1.00+0.10	30	1.35+0.27	7
Jia Wu(2)	2013	Asian	plasma	ACS	40	1.00+0.10	30	1.57+0.56	
Jia Wu(3)	2013	Asian	plasma	SAP	40	1.00+0.10	30	1.35+0.27	
Guofu Zhu®	2014	Asian	plasma	CAD	54	2.73±1.70	56	1.05±0.81	6
Guofu Zhu(2) ®	2014	Asian	PBMCs	CAD	54	2.09±0.95	56	1.12±0.86	
Guofu Zhu(3) ®	2014	Asian	plasma	Double-vessel disease	37	2.91±1.94	27	1.32±0.88	
Guofu Zhu(4) ®	2014	Asian	plasma	Three-vessel disease	37	2.91±1.94	31	0.83±0.66	
Ningshi Li	2014	Asian	PBMCs	AMI	42	1.43+0.553	38	0.51+0.089	6
Ningshi Li(2)	2014	Asian	PBMCs	UAP	42	1.43+0.553	33	0.75+0.246	
Ningshi Li(3)	2014	Asian	PBMCs	SAP	42	1.43+0.553	35	1.13+0.438	
Ningshi Li(4)	2014	Asian	PBMCs	CAD	58	1.24+0.732	90	0.62+0.547	
Ningshi Li(5)	2014	Asian	PBMCs	Double-vessel disease	58	1.24+0.732	38	0.83+0.375	
Ningshi Li(6)	2014	Asian	PBMCs	Three-vessel disease	58	1.24+0.732	36	0.73+0.335	
Ningshi Li(7)	2014	Asian	PBMCs	Mild stenosis	58	1.24+0.732	18	1.42+0.345	
Ningshi Li(8)	2014	Asian	PBMCs	Moderate stenosis	58	1.24+0.732	33	0.74+0.231	
Ningshi Li(9)	2014	Asian	PBMCs	Severe stenosis	58	1.24+0.732	39	0.69+0.247	
Qiang Su	2014	Asian	PBMCs	UAP	32	0.26±0.07	32	0.47±0.08	6
Qiang Su(2)	2014	Asian	PBMCs	CAD	32	0.26+0.07	32	0.47+0.08	
Qiang Su(3)	2014	Asian	PBMCs	Moderate stenosis	32	0.26±0.07	9	0.49±0.09	
Qiang Su(4)	2014	Asian	PBMCs	Severe stenosis	32	0.26±0.07	5	0.61±0.08	
Haitao Jiang	2017	Asian	PBMCs	CAD	208	0.84+0.37	200	1.43+0.73	5
Yuhua Xia	2017	Asian	plasma	CAD	16	1.00+0.14	16	0.85+0.22	6
Duo Zhao®	2018	Asian	plasma	AMI	26	3.00±0.80	21	0.69±0.10	5
Duo Zhao(2) ®	2018	Asian	plasma	SAP	26	3.00+0.80	23	1.55+0.29	
Duo Zhao(3) ®	2018	Asian	PBMCs	AMI	26	2.32+0.53	21	0.56+0.08	
Duo Zhao(4) ®	2018	Asian	PBMCs	SAP	26	2.32+0.53	23	1.34+0.25	
Xianke Qiu®	2018	Asian	plasma	CAD	100	1.11±0.442*	300	1.71±0.752*	6
Ziliang Ye®	2018	Asian	PBMCs	CAD	124	0.23+0.09	128	0.49+0.08	5
Ziliang Ye(2) ®	2018	Asian	PBMCs	Mild stenosis	124	0.23 ± 0.09	68	0.39 ± 0.12	
Ziliang Ye(3) ®	2018	Asian	PBMCs	Moderate stenosis	124	0.23 ± 0.09	38	0.54 ± 0.14	
Ziliang Ye(4) ®	2018	Asian	PBMCs	Severe stenosis	124	0.23 ± 0.09	22	0.68 ± 0.15	
Ziliang Ye(5) ®	2018	Asian	PBMCs	UAP	124	0.23 ± 0.09	128	0.49 ± 0.08	
Deyou Zhang	2019	Asian	plasma	Gensini ≤20	13	1.0915±0.192*	31	0.34±0.61*	5
Deyou Zhang(2)	2019	Asian	plasma	Gensini >20	13	1.0915±0.192*	11	0.07±0.134*	
Guofu Zhu®	2019	Asian	plasma	CAD	107	1.94±0.08	203	1.16±0.05	6
Guofu Zhu(2) ®	2019	Asian	PBMCs	CAD	107	2.37±0.09	203	1.46±0.05	
Guofu Zhu(3) ®	2019	Asian	PBMCs	Plaque stabilization	107	2.37±0.09	48	3.15±1.23	
Guofu Zhu(4) ®	2019	Asian	PBMCs	Plaque instability	107	2.37±0.09	52	2.79±1.05	
Guofu Zhu(5) ®	2019	Asian	plasma	Plaque stabilization	107	1.94±0.08	48	2.88±1.02	
Guofu Zhu(6) ®	2019	Asian	plasma	Plaque instability	107	1.94±0.08	52	2.12±0.89	
Kazimierczyk	2019	non-Asian	plasma	CAD	18	8.4475±2.091*	18	25.514±2.957*	6
Meiping Lin	2019	Asian	plasma	CAD	43	1.00+0.04	115	0.72+.011	7
Min Jia	2019	Asian	plasma	Plaque stabilization	50	0.83±0.11	50	0.71±0.09	7
Min Jia(2)	2019	Asian	plasma	Plaque instability	50	0.83+0.11	70	0.57+0.10	
Zhentao Lu	2019	Asian	PBMCs	CAD	50	0.89±0.384*	285	1.75±0.492*	6
Zhentao Lu(2)	2019	Asian	PBMCs	Single-vessel disease	50	0.89±0.384*	112	1.02±0.18	
Zhentao Lu(3)	2019	Asian	PBMCs	Double-vessel disease	50	0.89±0.384*	97	1.63±0.24	
Zhentao Lu(4)	2019	Asian	PBMCs	Three-vessel disease	50	0.89±0.384*	76	2.14±0.20	
Zhentao Lu(5)	2019	Asian	PBMCs	Mild stenosis	50	0.89±0.384*	88	1.47±0.24	
Zhentao Lu(6)	2019	Asian	PBMCs	Moderate stenosis	50	0.89±0.384*	146	1.70±0.21	
Zhentao Lu(7)	2019	Asian	PBMCs	Severe stenosis	50	0.89±0.384*	51	1.89±0.18	
Gang Li	2020	Asian	plasma	CAD	57	0.82+0.13	57	0.64+0.09	8

(1)Data with "*" is converted by a certain methods [18]

(2)The studies with "®" are English literature

(3)PBMCs, peripheral blood mononuclear cells; CAD, coronary artery disease; SAP, stable angina pectoris; ACS, acute coronary syndrome; AMI, acute myocardial infarction; UAP, unstable angina pectoris

### 3.3. Meta-analysis of circulating microRNA-155 in patients with and without CAD

#### 3.3.1 Comparison of microRNA-155 levels

**Fig 2:**
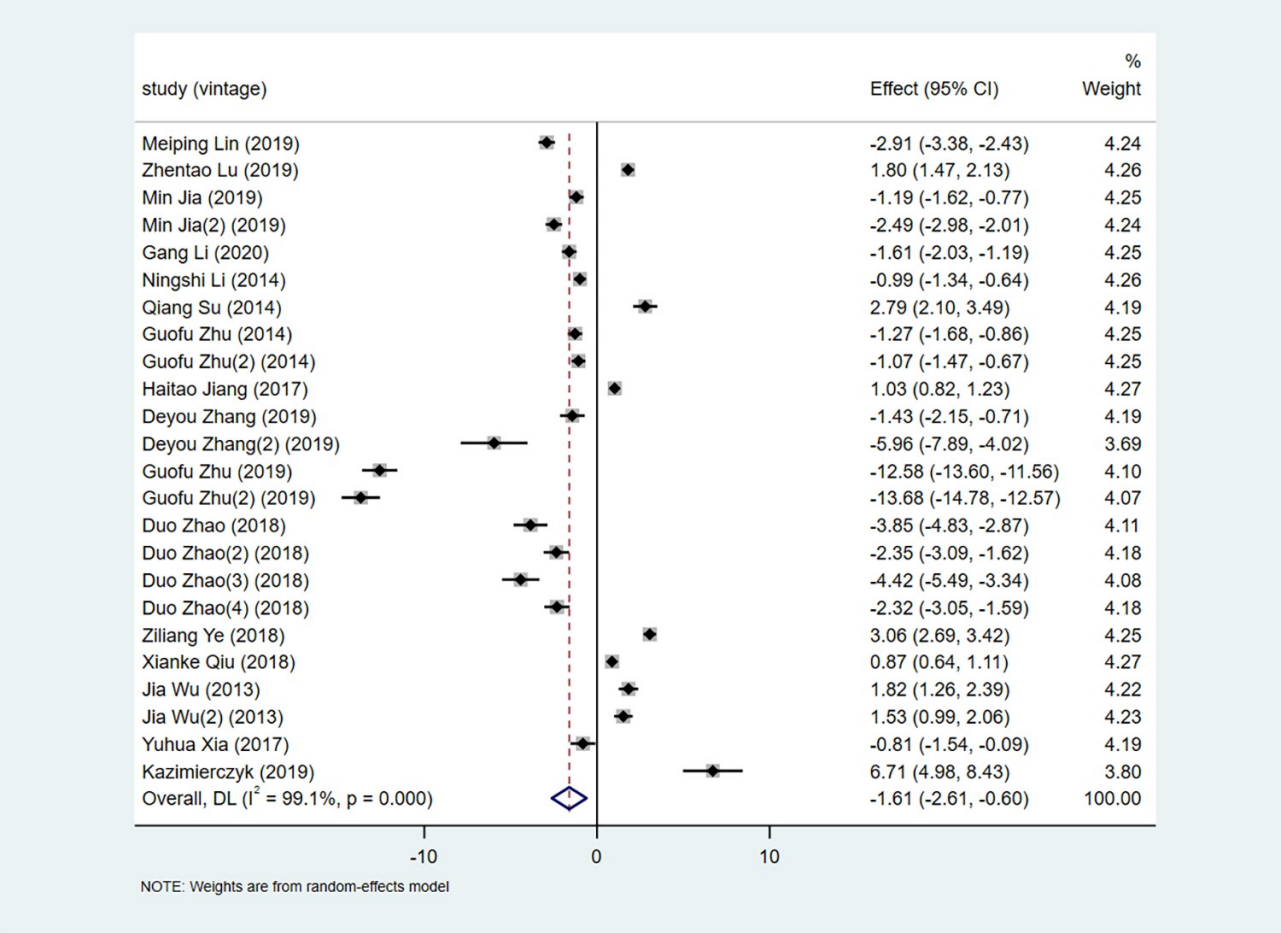
Forest plot of microRNA-155 association with CAD.

Meta-analysis of the association between expression of *microRNA-155* and CAD is shown in **Fig 2**. The results showed that the mean expression of *microRNA-155* was significantly lower in patients with CAD than in controls, with an SMD (95% CI) of -1.61 (-2.61, -0.60). The heterogeneity *I*^*2*^ was 99.1%, suggesting high heterogeneity. Meta-regression analysis was used to explore the source of heterogeneity. Covariates included race, sample size and sample source. However, meta-regression analysis did not find the cause of heterogeneity.

#### 3.3.2 Subgroup analyses

We attempted to explain the observed heterogeneity by assessing certain study characteristics, including subtypes of CAD, severity of CAD, sample source, sample size, and ethnicity, through subgroup analyses. As shown in **Table 2**, different types of CAD might be one of the source of heterogeneity.

**Table 2:** Results of subgroup analysis.

	Datasets	cases	controls	SMD(95%CI)	*p*	*I* ^*2*^	heterogeneity between groups(*P*)
**Sample source**							
plasma	15	1031	653	-1.70(-2.95, -0.44)	<0.001	98.80%	0.834
PBMCs	9	1038	685	-1.47(-3.17,0.24)	<0.001	99.30%	
**Sample size**							
N>100	7	545	364	-3.12(-5.39, -0.85)	<0.001	99.70%	0.084
N<100	17	1524	947	-0.99(-1.81, -0.18)	<0.001	97.10%	
**Ethnicity**							
Asian	23	2051	1320	-1.94(-2.95, -0.92)	<0.001	99.10%	<0.001
non-Asian	1	18	18	6.71(4.98,8.43)		100%	
**Subtype of CAD**							
AMI	3	80	94	-3.45(-4.86, -2.05)	<0.001	87.40%	0.005
UAP	3	193	198	1.44(-1.59,4.47)	<0.001	99.10%	
SAP	4	111	134	-0.85(-2.71,1.01)	<0.001	97.40%	
**Plaque stability**							
Plaque stabilization	6	146	364	0.53(-1.10,2.17)	<0.001	98.10%	0.404
Plaque instability	6	174	264	-0.47(-2.18,1.24)	<0.001	98.40%	
**Degree of coronary stenosis**							
mild stenosis	4	192	263	1.38(0.71,2.06)	<0.001	88.10%	0.544
moderate stenosis	4	226	263	2.06(-0.07,4.19)	<0.001	98.50%	
severe stenosis	4	117	263	2.91(-0.13,5.96)	<0.001	98.80%	
**Number of diseased vessels**							
single-vessel disease	3	143	144	0.22(-0.20,0.64)	>0.001	54.40%	0.956
double-vessel disease	3	162	144	0.28(-1.93,2.48)	<0.001	98.50%	
three-vessel disease	3	143	144	0.70(-2.46,3.87)	<0.001	99.10%	

Note: PBMCs, peripheral blood mononuclear cells; CAD, coronary artery disease; SAP, stable angina pectoris; AMI, acute myocardial infarction; UAP, unstable angina pectoris

When subgroup analysis by subtype of CAD was performed (**Table 2**), the results suggested that the level of *microRNA-155* was significantly lower in AMI patients than in controls (SMD = -3.45, 95% CI: -4.86, -2.05, *I*^*2*^ = 87.4%), though there was no significant difference in other subtypes of CAD. Regarding the severity of CAD (**Table 2**), the level of *microRNA-155* was significantly higher in CAD patients with mild stenosis than in controls (SMD = 1.38, 95% CI: 0.71, 2.06, *I*^*2*^ = 88.1%).

Subsequently, we conducted subgroup analysis by sample source (**Table 2**). The results indicated that the mean level of *microRNA-155* in the plasma of CAD patients was significantly lower than that in controls (SMD = -1.70, 95% CI: -2.95, -0.44, *I*^*2*^ = 98.8%). However, in the PBMCs of CAD patients, the level was not significantly different from that of controls (SMD = -1.47, 95% CI: -3.17, 0.24, *I*^*2*^ = 99.3%).

Subgroup analysis by sample size (**Table 2**) showed that in studies with large (N > 100) and small (N < 100) sample sizes, the level of *microRNA-155* in CAD patients was lower than that in controls. The SMD was lower in studies with large sample sizes than in studies with small sample sizes (-3.12 versus -0,99), suggesting that large-cohort studies are necessary and worthwhile.

#### 3.3.3 Sensitivity analysis

**Fig 3:**
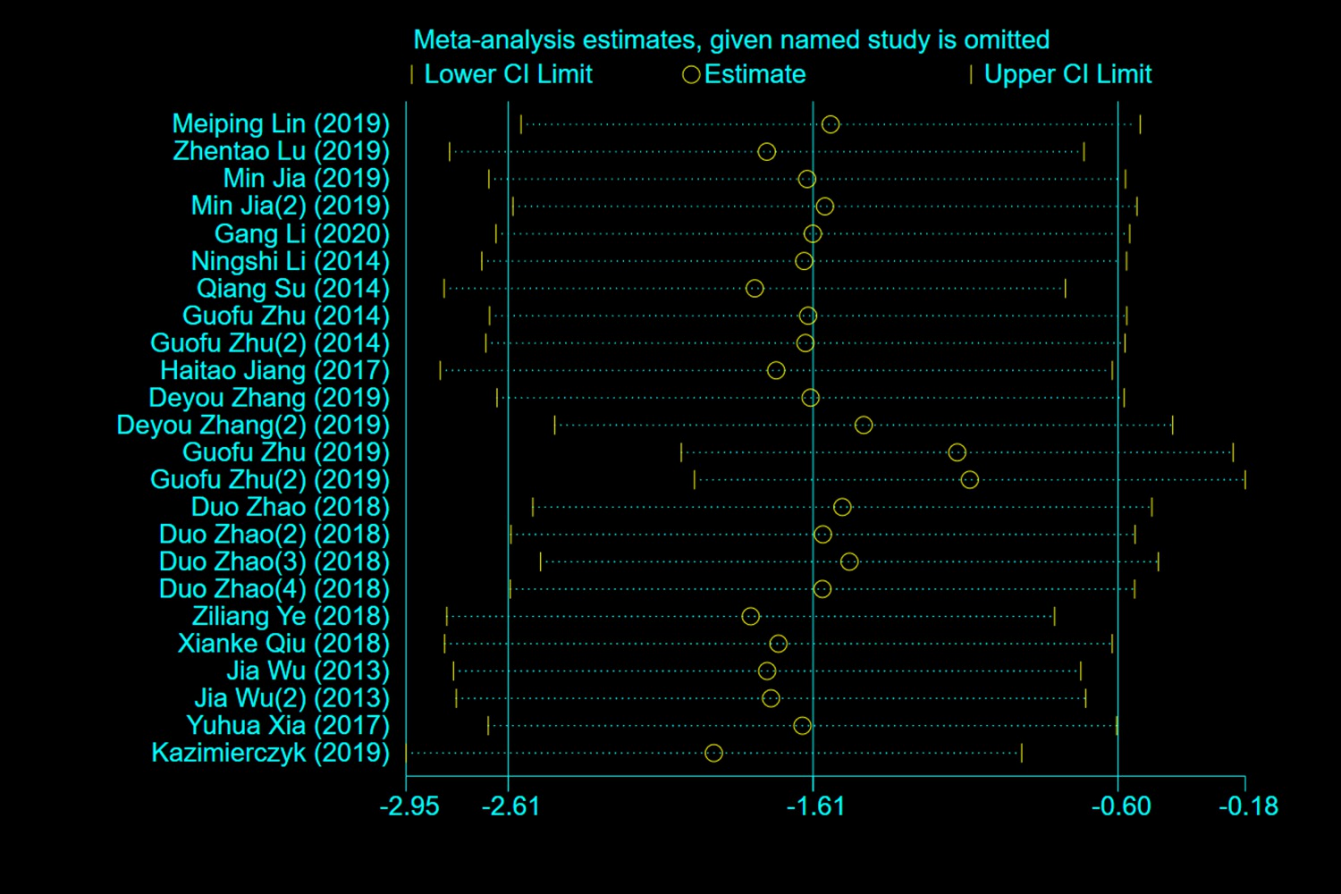
Sensitivity analysis.

In view of the high heterogeneity among the included studies (*I*^*2*^ = 99.1%), we subsequently performed sensitivity analysis (**Fig 3**). The results showed that removing any study had little effect on the combined results, suggesting the stability of the overall results.

#### 3.3.4 Publication bias

**Fig 4:**
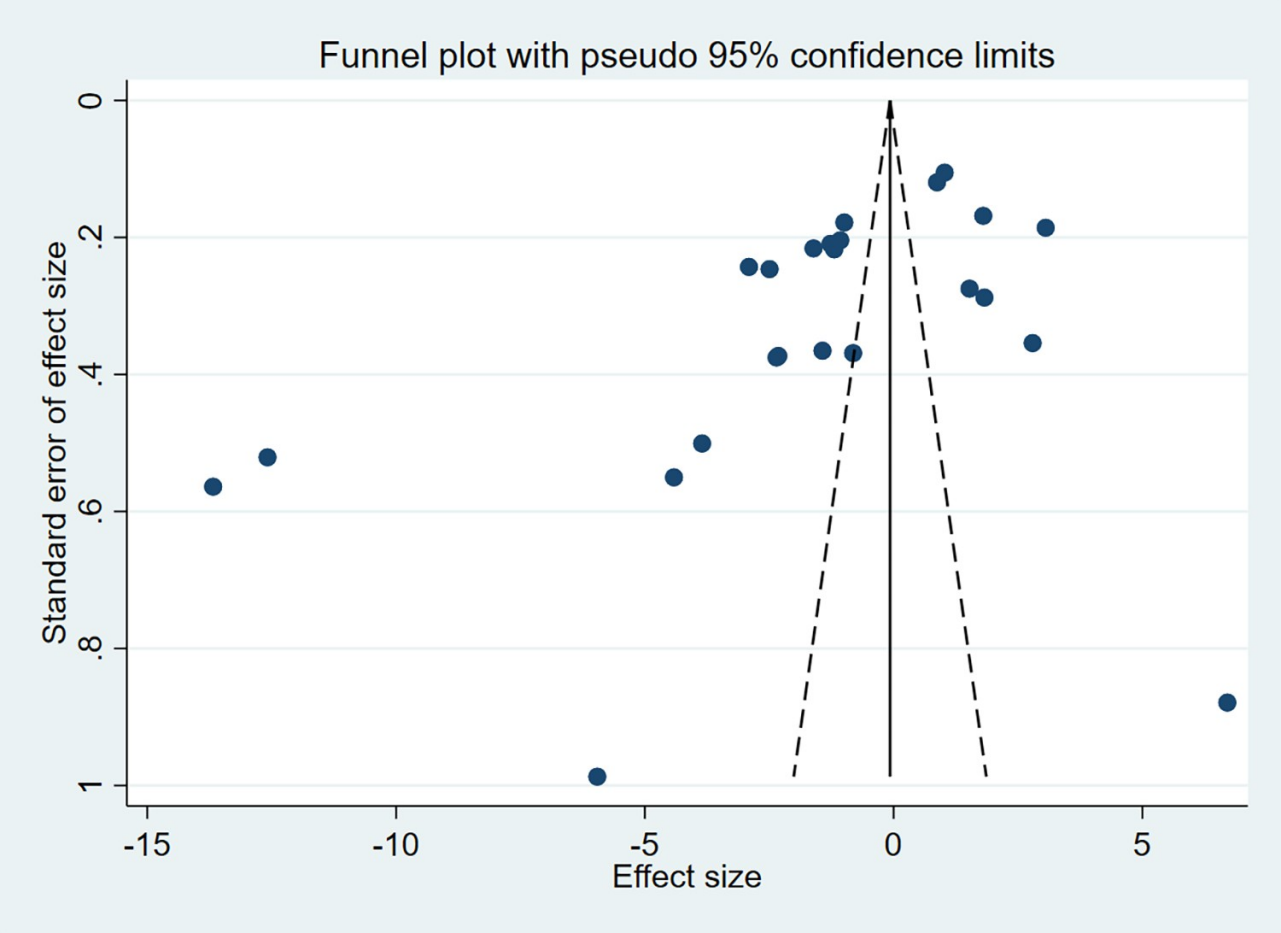
Funnel plots.

**Fig 5:**
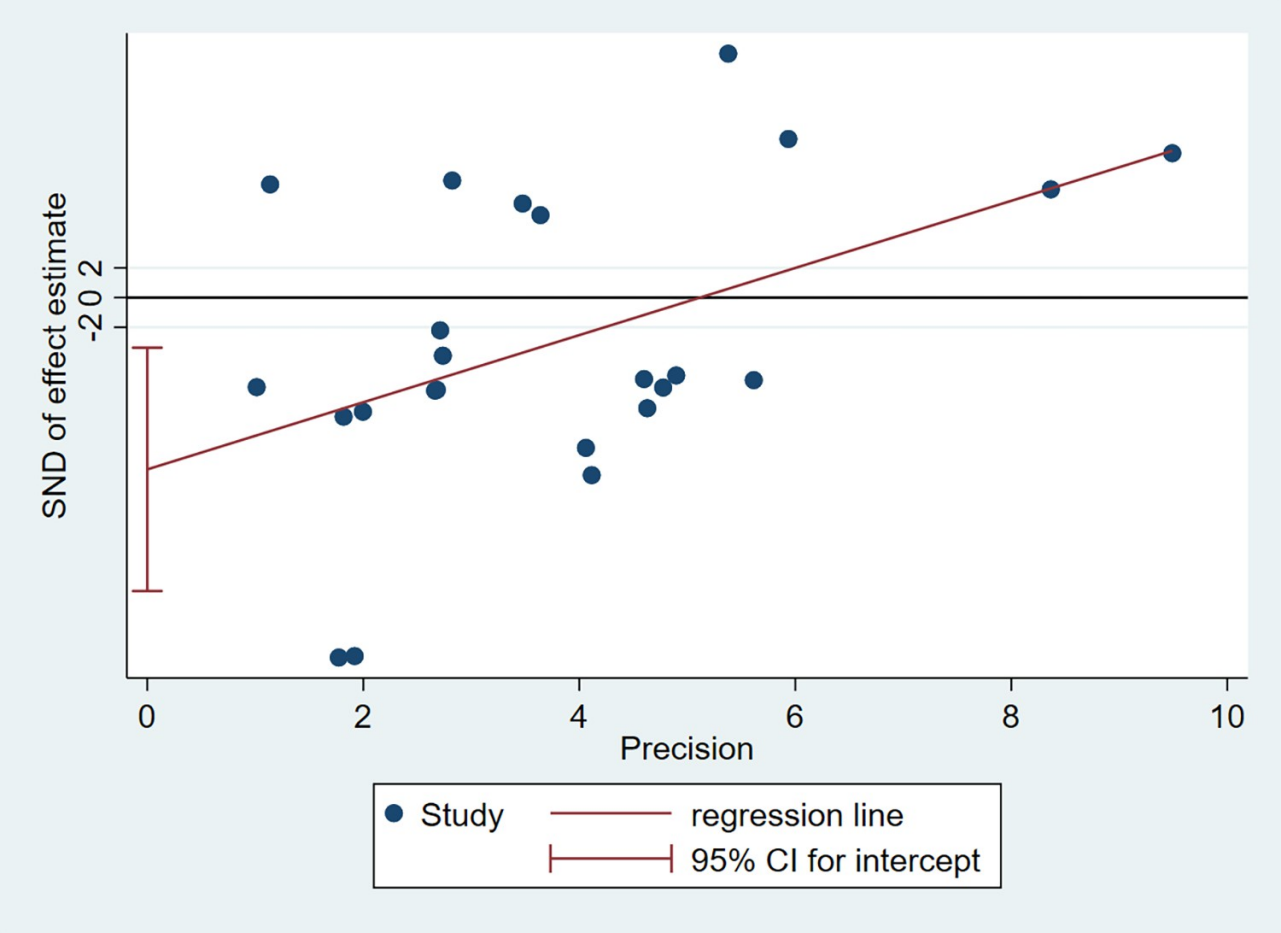
Egger test.

**Fig 6:**
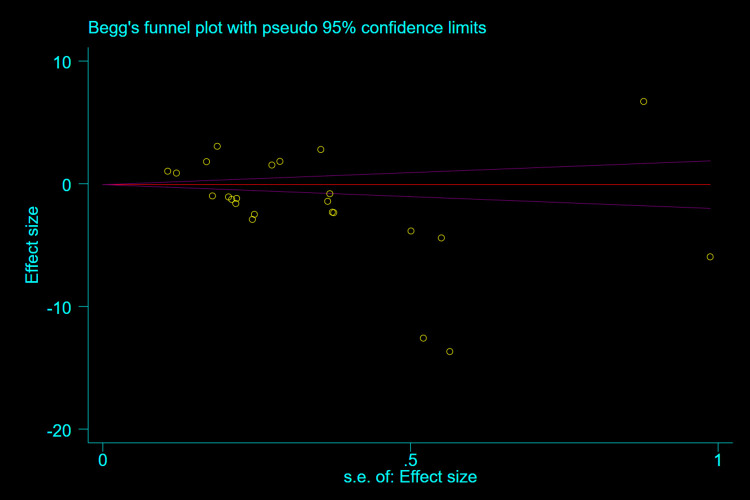
Begg’s test.

As shown in **Fig 4**, funnel plots were asymmetric, suggesting a certain publication bias. Egger’s test (P = 0.008, **Fig 5**) and Begg’s test (P = 0.011, **Fig 6**) also showed potential small-study bias and publication bias.

## 4 Discussion

To the best of our knowledge, this study is the first meta-analysis to comprehensively evaluate the level of *microRNA-155* in patients with CAD. Our meta-analysis, which included 16 articles with 3407 participants, showed that the mean level of *microRNA-155* was significantly lower in patients with CAD than in controls. Subgroup analyses suggested that the level of *microRNA-155* in the plasma of CAD patients and in acute myocardial infarction (AMI) patients was significantly lower than that in controls but that the level in CAD patients with mild stenosis was significantly higher than that in controls. We compared the three studies in the AMI subgroup strictly according to the PICOS method and found certain clinical heterogeneity, which may have been caused by different sampling times, different treatments of outcome indicators and different reagents in the process of RNA extraction. We adopted the combined fold change method to reduce related effects as much as possible, and many studies have confirmed the conclusion that the level of *microRNA-155* in AMI is lower than that in healthy individuals. Yu-Huizhang et al. showed that the plasma *microRNA-155* level in AMI patients is lower than that in CPS patients. Moreover, the expression level of *microRNA-155* in PBMCs correlated positively with the plasma level, indicating that the level of *microRNA-155* in PBMCs of AMI patients is lower than that of CPS patients (PMID: 25902164). In addition, Rui Yao et al.’s study found that AMI patient PBMCs had lower *microRNA-155* levels than CPS patient PBMCs (PMID: 21804579). These studies support our conclusion and add credibility to our results.

Numerous studies have indicated that aberrant *microRNA-155* expression is associated with tumor development [19–21]. As tumors and CAD share some risk factors and pathogenesis, many studies investigating the role of *microRNA-155* in CAD have been published in recent years. For the first time, our meta-analysis showed that expression of *microRNA-155* in CAD patients is lower than that in healthy individuals. The exact mechanism of *microRNA-155* in CAD remains unclear. Yunling Liu et al. [22] showed that *microRNA-155* inhibits translation of the target protein MyD88 through the Toll-like receptor pathway, thus preventing inflammatory factor release and inhibiting atherosclerosis development. In addition, Nazarijahantigh et al. [23] detected specific expression of *microRNA-155* in atherosclerotic plaques and macrophages, promoting atherosclerosis by inhibiting expression of the mouse target gene B-cell lymphoma 6 protein. Yulan Ma et al. [24] also found increased expression of *microRNA-155* in oxLDL-activated RAW264.7 cells and that increased *microRNA-155* induced expression of cell surface molecules (including MHC-I, MHC-II, CD86, CD83 and CD36) and secretion of cytokines (IL-6, IL-12 and IL-1B). Upregulation of these factors can promote the development of atherosclerosis (AS). These results indicate that the mechanism may be related to the regulation of inflammation and immune-related gene expression by *microRNA-155*.

In 1979, the World Health Organization recommended classifying CAD into five types, including asymptomatic ischemia, angina, myocardial infarction (MI), ischemic cardiomyopathy, and sudden death. It has been shown that plasma *microRNA-155* expression may also differ in patients with different types of coronary artery disease. Thus, in our study, we performed subgroup analysis based on subtypes of CAD. Consistent with the lower expression of *microRNA-155* in total CAD, we found that *microRNA-155* levels were significantly lower in AMI patients than in controls. The severity of CAD is related to the degree of stenosis, stability of plaques, and number of diseased vessels. Duo Zhao et al. found that expression of *microRNA-155* correlated highly negatively with the severity of coronary lesions. Conversely, our meta-analysis found that this level was significantly higher in CAD patients with mild stenosis than in healthy individuals. The reason for this difference is unknown, but a relatively small sample size may be involved. Further studies with larger sample sizes are needed to verify associations between *microRNA-155* and CAD or its subtypes.

In addition to *microRNA-155* as a predictor of CAD and tumors, evidence has shown that it can be used as a prognostic factor. A meta-analysis by Shufang Ning et al. reported that *microRNA-155* is a potential prognostic marker for the clinical management of hepatocellular carcinoma patients. Other studies also indicated that *microRNA-155* is a potential biomarker for predicting outcomes in various cancers, such as lung cancer, breast cancer, and glioma [21, 25–27]. However, there are few studies on the prognostic role of *microRNA-155* in CAD. Sen Matsumoto et al. found that circulating *microRNA-155* might be predictive for cardiac death in post-AMI patients [28], suggesting a broad prospect of *microRNA-155* in CAD. Further studies on the correlation between *microRNA-155* and the diagnosis and prognosis of CAD are necessary.

Most of the current clinical treatment regimens for CAD involve medication and surgery. However, drug therapy can only control the disease to a certain extent and alleviate symptoms, and surgery (such as PCI) is invasive, with a risk of recurrence. In recent years, gene-targeted therapy has also played a certain role in clinical practice (such as the use of gene knockout technology to prevent or treat breast cancer [29]), with great advantages over traditional treatment. Recep Bayraktar et al. suggested the potential of *microRNA-155*-based therapeutic approaches for the treatment of tumors [30]. Robyn Bruen et al. showed that inhibition of macrophage-specific *microRNA-155* might be a viable therapeutic strategy to reduce inflammation associated with atherosclerosis [31]. This study systematically showed decreased expression of *microRNA-155* in patients with CAD, which provides a basis for further study on the effect of *microRNA-155* in this disease. These data also suggest that targeting *microRNA-155* might become a hot research topic in the treatment of CAD in the future.

There are some limitations in this study. First, there were limitations in the data analysis due to some inaccessible literature or missing data, though we systematically searched eight databases. Second, the nonresponse rate was not described in the literature quality assessment; however, the scores of the included studies were more than five, which met the requirements of the meta-analysis. Third, there is no SD of the original mean in some of the literature in the data extraction, which led to the manual transformation of some of the data included in the study through certain methods; this may also result in a certain bias. Fourth, there was high heterogeneity and bias, even though sensitivity analysis suggested a relatively stable result. High heterogeneity may influence the sensitivity and specificity of results, and subgroup analyses showed that different types of CAD might be one of the sources of heterogeneity. The existence of publication bias might be related to the elimination of many foreign studies due to the unavailability of data. Fifth, differences caused by the sample population, primer manufacturers and batches and operators, detection instruments and kits are inevitable. Obviously, such differences may be a source of heterogeneity. Unfortunately, as we could not obtain this information from the original articles, we could not perform subgroup analysis for heterogeneity detection. Nevertheless, the meta-analysis included a large sample of the literature; heterogeneity between studies could not be completely avoided, and publication bias is naturally present in a meta-analysis. Further studies with larger samples and higher quality are needed to confirm the association between *microRNA-155* and CAD.

## 5 Conclusion

In conclusion, our study showed for the first time through meta-analysis that the expression level of circulating *microRNA-155* in patients with CAD is lower than that in non-CAD although. Our study highlights the important role of *microRNA-155* in different types and stages of clinical CAD, provides a new possible reference index for the diagnosis and monitoring of patients with CAD, and provides data support for the treatment of these patients.

## Supporting information

S1 File PCR details. (XLSX)

S2 File PRISMA_2020_checklist. (DOCX)

S3 File Retrieval strategy. (DOCX)
