# Supplementary material for: A meta-analysis of the relationship between circulating microRNA-155 and coronary artery disease
Source: PLoS One. 2023 Apr 13;18(4):e0274277. doi: 10.1371/journal.pone.0274277 (PMC10101406; doi:10.1371/journal.pone.0274277)
Supplement: S3 File — (DOCX) [file pone.0274277.s003.docx]

**S3 Table. Retrieval strategy**

| **Database** | **Keywords** | **N** |
| --- | --- | --- |
| Web of Science | (“Coronary heart disease”OR“CHD”OR“coronary artery disease”OR“coronary disease”OR“CAD”OR“heart  disease”OR“coronary  atherosclerosis”OR“angina”OR“myocardial  infarction”OR“ischemic  heart  disease”OR“acute  coronary  syndrome”OR“ischemic  heart  failure”OR“ischemic cardiomyopathy”) AND (“microRNA-155” OR  “miR-155”  OR  “miRNA-155”) | 297 |
| Embase | (“Coronary heart disease”OR“CHD”OR“coronary artery disease”OR“coronary disease”OR“CAD”OR“heart  disease”OR“coronary  atherosclerosis”OR“angina”OR“myocardial  infarction”OR“ischemic  heart  disease”OR“acute  coronary  syndrome”OR“ischemic  heart  failure”OR“ischemic cardiomyopathy”) AND (“microRNA-155” OR  “miR-155”  OR  “miRNA-155”) | 239 |
| Google  Scholar | (“Coronary heart disease”、“CHD”、“coronary artery disease”、“coronary disease”、“CAD”、“heart  disease”、“coronary  atherosclerosis”、“angina”、“myocardial  infarction”、“ischemic  heart  disease”、“acute  coronary  syndrome”、“ischemic  heart  failure”、“ischemic cardiomyopathy”) AND (“microRNA-155” 、  “miR-155”  、  “miRNA-155”) | 23 |
| Cochrane Library | (“Coronary heart disease”、“CHD”、“coronary artery disease”、“coronary disease”、“CAD”、“heart  disease”、“coronary  atherosclerosis”、“angina”、“myocardial  infarction”、“ischemic  heart  disease”、“acute  coronary  syndrome”、“ischemic  heart  failure”、“ischemic cardiomyopathy”) AND (“microRNA-155” 、  “miR-155”  、  “miRNA-155”) | 6 |
| PubMed | (microRNA-155[Title/Abstract] OR miR-155 [Title/Abstract] OR miRNA-155[Title/Abstract]) AND (Coronary heart disease[Title/Abstract] OR CHD[Title/Abstract] OR coronary artery disease[Title/Abstract] OR coronary disease[Title/Abstract] OR CAD [Title/Abstract] OR heart disease [Title/Abstract] OR coronary atherosclerosis [Title/Abstract] OR angina [Title/Abstract] OR myocardial infarction [Title/Abstract] OR ischemic heart disease [Title/Abstract] OR acute coronary syndrome [Title/Abstract] OR ischemic heart failure [Title/Abstract] OR ischemic cardiomyopathy[Title/Abstract]) | 79 |
| Chinese National Knowledge Infrastructure (CNKI) | 冠心病、冠状动脉疾病、心肌梗塞、缺血性心脏病、缺血性心血管病、microRNA-155、miR-155、miRNA-155、微小RNA-155、微小核糖核酸-155 | 118 |
| Wanfang Database | 冠心病、冠状动脉疾病、心肌梗塞、缺血性心脏病、缺血性心血管病、microRNA-155、miR-155、miRNA-155、微小RNA-155、微小核糖核酸-155 | 34 |
| China Science and Technology Journal Database (VIP) | 冠心病、冠状动脉疾病、心肌梗塞、缺血性心脏病、缺血性心血管病、microRNA-155、miR-155、miRNA-155、微小RNA-155、微小核糖核酸-155 | 16 |
